# Toll-like receptor 4-mediated cytokine synthesis and post-stroke depressive symptoms

**DOI:** 10.1038/s41398-021-01359-x

**Published:** 2021-04-26

**Authors:** Michal Korostynski, Dzesika Hoinkis, Marcin Piechota, Slawomir Golda, Joanna Pera, Agnieszka Slowik, Tomasz Dziedzic

**Affiliations:** 1 Department of Molecular Neuropharmacology, Maj Institute of Pharmacology, Polish Academy of Sciences, Krakow, Poland; 2 Intelliseq sp. z o.o, Krakow, Poland; 3 Department of Neurology, Jagiellonian University Medical College, Krakow, Poland

**Keywords:** Depression, Pathogenesis

## Abstract

Altered cytokine synthesis thought to contribute to the pathophysiology of post-stroke depression (PSD). Toll-like receptor 4 (TLR4) is a master regulator of innate immunity. The aim of this study was to explore the putative association between TLR4-mediated cytokine synthesis and subsequent symptoms of PSD. In total, 262 patients with ischemic stroke and without a history of PSD were included. Depressive symptoms were assessed using the Patient Health Questionnaire-9 in 170 patients on Day 8 and in 146 at 3 months after stroke. Blood samples taken on Day 3 after stroke were stimulated ex vivo with lipopolysaccharide (LPS). Ex vivo synthesized cytokines (TNFα, IP-10, IL-1β, IL-6, IL-8, IL-10, and IL-12p70) and circulating cytokines (TNFα, IL-6, sIL-6R, and IL-1ra) were measured using the enzyme-linked immunoassay or cytometric method. RNA sequencing was used to determine the gene expression profile of LPS-induced cytokines and chemokines. LPS-induced cytokine synthesis and the gene expression of TLR4-dependent cytokines and chemokines did not differ between patients with and without greater depressive symptoms. The plasma level of IL-6, but not TNFα, sIL-6R, and IL-1ra, was higher in patients who developed depressive symptoms at 3 months after stroke (median: 4.7 vs 3.4 pg/mL, *P* = 0.06). Plasma IL-6 predicted the severity of depressive symptoms at 3 months after stroke (β = 0.42, *P* = 0.03). In conclusion, TLR4-dependent cytokine synthesis was not associated with greater post-stroke depressive symptoms in this study. Circulating IL-6 might be associated with depressive symptoms occurring at 3 months after stroke.

## Introduction

A substantial amount of evidence has shown that cytokine-mediated communication between the immune system and brain is involved in the pathogenesis of depressive symptoms^1,2^. First, patients with major depressive disorder (MDD) have higher levels of circulating cytokines than nondepressed persons^3–5^. Of the various cytokines, the best replicated association with depression has been found for plasma interleukin-6 (IL-6), and to a lesser degree, IL-1β and tumor necrosis factor alpha (TNFα). Furthermore, a longitudinal study showed that higher serum IL-6 levels in childhood predated the occurrence of depression in young adulthood^6^. Second, the increased expression of genes related to IL-6, IL-8, and type I interferon (INF)-induced signaling pathways has been found in blood cells of patients with depression^7,8^. Third, the administration of cytokines (e.g., INFα) or inducers of cytokines such as endotoxin or typhoid vaccination triggers depressive symptoms, even in healthy individuals^9–11^. Fourth, anticytokine therapy with monoclonal antibodies or cytokine inhibitors leads to improvement in depressive symptoms^12^. Fifth, animal studies have shown that circulating cytokines can enter the brain and have been implicated in the pathogenesis of depression, including neurotransmission, neurogenesis, neurotrophic support, and neuroendocrine functions^13^.

Post-stroke depression (PSD) affects approximately one-third of stroke survivors and is associated with significant disability, mortality, and cognitive impairment^14,15^. PSD seems to be multifactorial in origin. As with idiopathic depression, the cytokine hypothesis has been proposed to explain the pathogenesis of PSD^16^. This hypothesis assumes that increased synthesis of proinflammatory cytokines in stroke patients triggers depression by interfering with neurotransmission and activity of the hypothalamus-pituitary-adrenal axis. However, to date, there is no definitive evidence supporting this hypothesis. Clinical studies measuring circulating cytokine levels in patients with PSD have yielded conflicting results. A few studies have found that increased serum concentrations of IL-1β^17^, IL-6^18–21^, IL-10^19^, IL-18^21,22^, TNFα^19^, and INFɣ^19^ are associated with PSD, whereas other studies have not confirmed these observations^18,23,24^.

Toll-like receptors (TLRs) are master regulators of cytokine synthesis^25^. The most studied member of this family is TLR4. TLR4 signaling in response to lipopolysaccharide (LPS) includes two distinct pathways^25^. The myeloid differentiation primary response 88 (MyD88)-dependent pathway triggers the expression of proinflammatory cytokine genes such as TNFα, whereas the MyD88-independent pathway leads to activation of type I INFs and INF-inducible chemokines such as interferon-gamma inducible protein 10 (IP-10)^25,26^.

The mRNA and protein expression of TLR4 is reportedly increased in the blood cells of patients with MDD^27^. Successful pharmacotherapy and psychotherapy reduces the peripheral activity of TLR4^28^.

There are several reasons to study TLR4-mediated cytokine synthesis in patients with PSD. First, in patients with ischemic stroke, TLR4 expression on monocytes increases and correlates with circulating inflammatory mediators and stroke severity^29^. Second, altered TLR4-mediated cytokine production, including reduced proinflammatory cytokine release and elevated synthesis of IL-10 and IL-8, has been found in patients with poor outcome after stroke^30^. Third, the modulation of TLR4-dependent cytokine synthesis might be a potential therapeutic target^31,32^. For example, exercise ameliorates PSD in mice by inhibiting TLR4-dependent downstream inflammatory signalling^33^.

To the best of our knowledge, ex vivo cytokine synthesis has not been investigated in the context of PSD.

The aim of this study was to explore the putative association between TLR4-mediated cytokine synthesis and subsequent symptoms of PSD.

## Methods

### Patient selection and clinical assessment

The participants in this study were prospectively recruited from consecutive stroke patients who were hospitalized in the Department of Neurology, University Hospital, Krakow, Poland, between October 2016 and December 2018. The inclusion criteria were: (1) ischemic stroke; (2) time from the onset of stroke symptoms to admission <24 h; (3) prestroke modified Rankin Scale score of 0–2 (independent of daily activities); (4) National Institute of Health Stroke Scale (NIHSS) score on admission >3; and (5) informed patient consent. The exclusion criteria were: (1) chronic inflammatory, autoimmune, or cancerous diseases; (2) the use of steroids or immunomodulatory drugs before stroke or in the acute phase of stroke; (3) prestroke diagnosis of MDD; and (4) the use of antidepressive drugs before stroke. In addition, patients who were treated with antidepressants after hospital discharge and had a lower score of depressive symptoms at 3 months after stroke were excluded. These patients were precluded from the analysis, because it was impossible to determine if a lower score of their depressive symptoms reflected the natural history of mood symptoms after stroke or was the effect of treatment. Written informed consent was obtained from each patient included in the study. The study protocol was approved by the Bioethics Committee of Jagiellonian University.

The presence of depressive symptoms was assessed on Day 8 ± 1 and at 3 months after stroke onset using the Patient Health Questionnaire-9 (PHQ-9)^34^. The PHQ-9 is a valid and clinically feasible depression screening tool for stroke^35^. Score ≥10 is considered indicative of greater depressive symptoms^36,37^. Consequently, patients were divided into two groups: with and without greater depressive symptoms. Before PHQ-9 administration, aphasia was examined using clinical methods. Patients who were not able to understand questions were excluded from the study.

Neurological deficit on admission was assessed using the NIHSS, which quantifies stroke-related neurological deficit. Higher scores indicate greater impairment and more severe stroke^38^.

Stroke etiology was determined using the TOAST criteria^39^.

The Informant Questionnaire on Cognitive Decline in the Elderly (IQCODE) with a cutoff of 3.3 was used to diagnose prestroke cognitive decline^40,41^. The IQCODE consists of 26 items that rate change in patients’ intellectual abilities over the past 10 years.

### Cytokine assays

The whole blood stimulation procedure was previously described^30^. Venous blood was collected in heparinized tubes (Sarstedt, Nuembrecht, Germany) on Day 3 after stroke. To avoid diurnal variation, blood was obtained between 7:00 AM and 7:30 AM. The whole blood was diluted by 1:5 in sterile RPMI 1640 medium supplemented with L-glutamine (Sigma–Aldrich, St. Louis, MO). Then the samples were stimulated in sterile tubes (Lonza, Walkersville, MD) at 37 °C in 5% CO_2_ with LPS (10 ng/mL, *E. coli* 0111:B4, Sigma–Aldrich, St. Louis, MO). Blood stimulation was performed for 4 h for TNFα and IP-10, and 24 h for IL-1β, IL-6, IL-8, IL-10, and IL-12p70. The supernatants were removed and stored at −80 °C until further analysis. TNFα and IP-10 concentrations were measured using a commercially available enzyme-linked imunoassay (ELISA) kit (R&D Systems, Minneapolis, MN) according to the manufacturer’s instructions. The levels of IL-1β, IL-6, IL-8, IL-10, and IL-12p70 were determined by a cytometric bead array (Human Inflammatory Kit, BD Biosciences, San Diego, CA).

Plasma levels of IL-6, soluble IL-6 receptor (sIL-6R), TNFα, and IL-1 receptor antagonist (IL-1ra) were measured using a commercially available ELISA kit (R&D Systems, Minneapolis, MN). The detection limit was 0.11 pg/mL for IL-6, 15.1 pg/mL for sIL-6R, 0.19 pg/mL for TNFα, and 18.3 pg/mL for IL-1ra.

### Bulk RNA-sequencing

After whole blood stimulation with LPS for 4 h, cell pellets were transferred to the PAXgene Blood RNA Tube (PreAnalytiX, GmbH, Switzerland) under sterile conditions and stored at −80 °C for subsequent RNA analysis.

Total RNA was isolated from blood using the PAXgene Blood RNA Kit (Qiagen, Valencia, CA) following the manufacturer’s protocol.

RNA concentrations were measured using the NanoDrop ND-1000 Spectrophotometer (NanoDrop Technologies, Montchanin, DE) and RNA quality was determined by chip-based capillary electrophoresis utilizing the Agilent RNA 6000 Pico Kit (Agilent, Santa Clara, CA) and Agilent Bioanalyzer 2100 (Agilent, Palo Alto, CA). Samples with poor quality RNA (RNA integrity number < 7) were excluded from gene expression profiling.

The mRNA libraries were generated with NEBNext Ultra II Directional RNA Library Prep Kit for Illumina (New England Biolabs, Ipswich, MA). The polyA transcriptome libraries were sequenced on NovaSeq 6000 (Illumina) sequencer with the following parameters: PE 150 (paired ends) and 40 M clean reads, which yielded more than 12 Gb of raw data per each sample.

The quality of next-generation sequencing data was verified using FastQC v. 0.11.8. The RNA-sequencing reads were aligned to GRCh38.p13 using Hisat2 v. 2.0.5. The transcript Fragments Per Kilobase of transcript, per Million mapped reads were quantified using Cufflinks v. 2.2.1 and General Transfer Format from the Ensembl gene database.

Raw data are publicly available at: https://dataview.ncbi.nlm.nih.gov/object/PRJNA707400?reviewer=dteesjlnv9h7lo8kv70b65aohj.

### Statistical analysis

#### Cytokine synthesis analysis

Since cytokine levels and some clinical parameters (age, NIHSS score) had not normal distribution, the Mann–Whitney *U*-test was used to compare continuous variables between groups. These data are shown as medians with interquartiles. The χ^2^ test was used to compare proportions. Linear regression was used to model the relationship between square root transformed cytokine values and depressive symptoms severity after exclusion of outliers. The calculations were performed using STATISTICA software (version 12.5, Statsoft, Poland).

Due to a lack of previously published studies on ex vivo cytokine synthesis in patients with PSD, which are necessary for power calculations, we did not compute a priori power analysis.

#### RNA-sequencing data analysis

Statistical significance was analyzed using one-way analysis of variance on log2(1 + x) values, which have normal continuous distribution. The assumption of homogeneity of variance has been checked. The false discovery rate was estimated using the Benjamini–Hochberg method. All statistical analyses were performed using R software v3.4.3. Transcript annotation and classification were performed using the BioMart interface for the Ensembl gene database.

Genes (232) included in the Gene Ontology category, “cellular response to lipopolysaccharide” (GO:0071222), were considered to be involved in LPS-mediated immune responses and analyzed for alterations in gene expression levels (http://amigo.geneontology.org/amigo/term/GO:0071222).

Moreover, based on previous studies^42–44^, cytokines and chemokines induced by TLR4 stimulation were selected, and their gene expression levels were compared between groups.

## Results

### Group characteristics

**Fig. 1:**
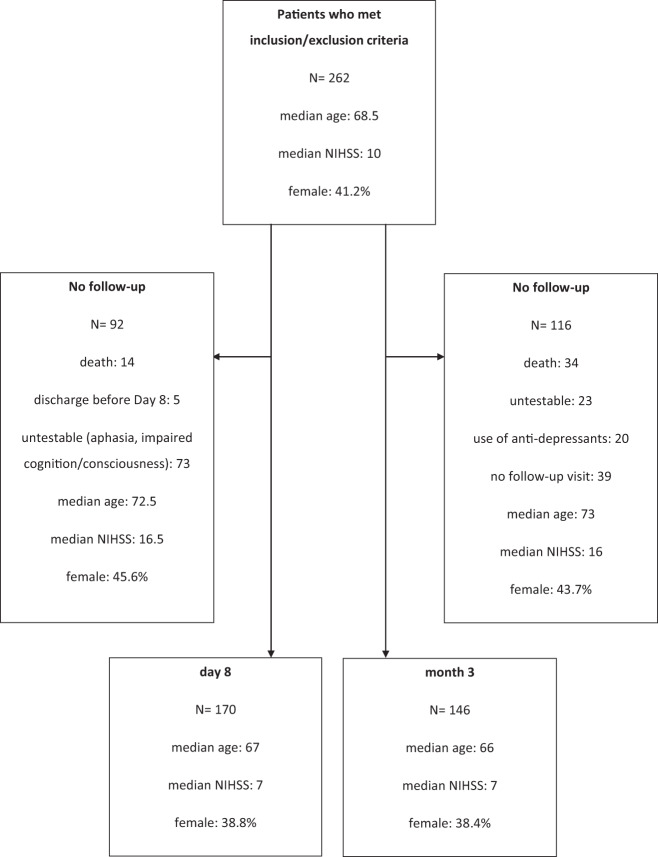
Excluded and included patients. NIHSS: National Institute of Health Stroke Scale. Flow chart showing the numbers of patients included in the study.

In total, 262 patients met the inclusion and exclusion criteria. Of them, 170 and 146 patients underwent assessment of depressive symptoms on Day 8 and at 3 months after stroke, respectively (Fig. 1).

Greater depressive symptoms were found in 15.9% of patients who underwent depressive symptoms assessment on Day 8 and in 25.3% patients who underwent depressive symptoms assessment at 3 months after stroke. Baseline characteristics of the groups are shown in Tables 1 and 2.

**Table 1:** Baseline characteristics of patients with high and patients with low depressive symptoms score on Day 8.

	High score of depressive symptoms (*N* = 27)	Low score of depressive symptoms (*N* = 143)	*P*-value
Age (years), median (IQs)	67 (58–73)	67 (61–78)	0.54
Female, *n* (%)	7 (25.9)	59 (41.3)	0.13
Hypertension, *n* (%)	21 (77.8)	107 (74.8)	0.74
Diabetes mellitus, *n* (%)	9 (33.3)	36 (25.2)	0.38
Atrial fibrillation, *n* (%)	10 (37.0)	40 (28.0)	0.34
Myocardial infarction, *n* (%)	4 (14.8)	16 (11.2)	0.60
Previous stroke or TIA, *n* (%)	3 (11.1)	15 (10.5)	0.92
Prestroke cognitive decline, *n* (%)^a^	5/22 (22.7)	9/118 (7.6)	0.03
NIHSS score on admission, *n* (%)	7 (4–13)	7 (5–14)	0.36
Stroke etiology			0.40
Large vessel disease, *n* (%)	5 (18.5)	34 (23.7)	
Small vessel disease, *n* (%)	0 (0)	10 (7.0)	
Cardio-embolic, *n* (%)	9 (33.3)	42 (29.4)	
Other, *n* (%)	0 (0)	5 (3.5)	
Undermined, *n* (%)	13 (48.2)	52 (36.4)	
Intravenous thrombolysis, *n* (%)	14 (51.8)	81 (56.6)	0.64
Mechanical thrombectomy, *n* (%)	4 (14.8)	39 (27.3)	0.17
In-hospital pneumonia, *n* (%)	3 (11.1)	7 (4.9)	0.21
White blood cells count, ×10^3^/µL, median (IQs)	8.7 (7.1–10.0)	7.8 (6.6–9.4)	0.13
Lymphocyte, ×10^3^/µL, median (IQs)	1.8 (1.5–2.3)	1.8 (1.4–2.2)	0.65
Monocyte, ×10^3^/µL, median (IQs)	0.7 (0.7–1.1)	0.8 (0.6–0.9)	0.44

^a^Data available for 140 patients.

**Table 2:** Baseline characteristics of patients with high and patients with low depressive symptoms score at 3 months.

	High score of depressive symptoms (*N* = 37)	Low score of depressive symptoms (*N* = 109)	*P*-value
Age (years), median (IQs)	66 (58–73)	66 (57–76)	0.95
Female, *n* (%)	11 (29.7)	45 (41.3)	0.21
Hypertension, *n* (%)	30 (81.1)	84 (77.1)	0.61
Diabetes mellitus, *n* (%)	8 (21.6)	30 (27.5)	0.48
Atrial fibrillation, *n* (%)	10 (27.0)	25 (22.9)	0.61
Myocardial infarction, *n* (%)	5 (13.5)	12 (11.0)	0.68
Previous stroke or TIA, *n* (%)	5 (13.5)	13 (11.9)	0.80
Prestroke cognitive decline^a^, *n* (%)	2/32 (6.2)	6/87 (6.9)	0.90
NIHSS score on admission, *n* (%)	9 (6–12)	7 (4–14)	0.57
Stroke etiology			0.75
Large vessel disease, *n* (%)	9 (24.3)	26 (23.8)	
Small vessel disease, *n* (%)	2 (5.4)	6 (5.5)	
Cardio-embolic, *n* (%)	9 (24.3)	28 (25.7)	
Other, *n* (%)	0 (0)	5 (4.6)	
Undermined, *n* (%)	17 (46.0)	44 (40.4)	
Intravenous thrombolysis, *n* (%)	20 (54.0)	64 (58.7)	0.62
Mechanical thrombectomy, *n* (%)	8 (21.6)	28 (25.7)	0.62
In-hospital pneumonia, *n* (%)	2 (5.4)	3 (2.7)	0.44
White blood cells count, ×10^3^/µL, median (IQs)	8.8 (6.9–10.1)	7.9 (6.7–9.0)	0.21
Lymphocyte, ×10^3^/µL, median (IQs)	2.0 (1.4–2.4)	1.8 (1.5–2.3)	0.52
Monocyte, ×10^3^/µL, median (IQs)	0.8 (0.7–0.9)	0.8 (0.6–0.90)	0.30

^a^Data available for 119 patients.

The groups did not significantly differ in age, sex, stroke severity, stroke etiology, and distribution of vascular risk factors. Patients with greater depressive symptoms on Day 8 more often suffered from prestroke cognitive impairment compared with patients without greater depressive symptoms (OR: 1.05–12.03, *P* = 0.04).

### Ex vivo synthesized and circulating cytokines

TLR4-mediated cytokine synthesis ex vivo did not differ between groups (Table 3).

**Table 3:** Ex vivo synthesized and circulating cytokines in stroke patients.

	Day 8			3 months		
	High score of depressive symptoms (*N* = 27)	Low score of depressive symptoms (*N* = 143)	*P*-value	High score of depressive symptoms (*N* = 37)	Low score of depressive symptoms (*N* = 109)	*P*-value
Ex vivo cytokines (pg/mL)						
TNFα	2551 (1596–3403)	2451 (1785–3573)	0.72	2665 (1824–3297)	2644 (1967–3891)	0.42
IL-1β	1399 (930–1964)	1573 (1101–2378)	0.22	1533 (1046–2141)	1657 (1120–2378)	0.38
IL-6	11168 (8000–18,211)	11802 (8203–17,057)	0.80	10822 (8000–16,527)	12427 (8853–17,177)	0.40
IL-8	1339 (944–2194)	1651 (1012–2548)	0.37	2037 (1143–2665)	1528 (956–2331)	0.16
IL-12	5.4 (0.6–10.3)	4.1 (0.3–8.6)	0.26	3.9 (2.4–6.9)	5.4 (0.7–9.8)	0.41
IL-10	44.9 (36.6–69.2)	51.8 (32.0–75.1)	0.62	46.2 (32.9–78.1)	44.9 (32.0–70.2)	0.38
IP-10	485 (251–821)	436 (197–793)	0.81	371 (164–627)	468 (273–775)	0.16
Plasma cytokines (pg/mL)						
IL-6	5.3 (2.0–19.4)	3.8 (2.0–7.3)	0.25	4.7 (2.9–12.0)	3.4 (1.8–7.1)	0.06
TNFα^a^	0.7 (0.4–0.9)	0.7 (0.4–1.1)	0.57	0.7 (0.4–1.1)	0.7 (0.3–1.1)	0.53
IL-1ra^b^	552 (324–814)	544 (362–895)	0.89	499 (353–809)	539 (362–946)	0.83
sIL-6R^c^	28,800 (26,800–34,600)	32,700 (26,000–38,000)	0.48	32,400 (26,350–40,150)	31,000 (25,700–35,500)	0.23

^a^Samples available for 123 (17/106) patients and 107 (27/80) patients who were examined on Day 8 and 3 months after stroke, respectively.

^b^Samples available for 127 (19/108) patients and 134 (36/98) patients who were examined on Day 8 and 3 months after stroke, respectively.

^c^Samples available for 138 (21/117) patients and 134 (36/98) patients who were examined on Day 8 and 3 months after stroke, respectively.

Plasma levels of IL-6, sIL-6R, TNFα, and IL-1ra did not differ between groups. A trend (*P* = 0.06) towards higher circulating level of IL-6 was observed after 3 months in patients with greater depressive symptoms.

Sex-stratified analysis has shown no differences in cytokine synthesis ex vivo in patients with and without greater depressive symptoms. However, males with depressive symptoms tended to have a higher circulating level of IL-6 (Supplementary Table 1).

Linear regression revealed the weak association between the reduced synthesis of IL-1β and IP-10 and severity of depressive symptoms assessed on Day 8 (Table 4). Plasma IL-6 predicted the severity of depressive symptoms assessed at 3 months after stroke. There was no association of age, sex, stroke severity, prestroke cognitive impairment, and vascular risk factors with severity of depressive symptoms.

**Table 4:** Associations of cytokines with severity of depressive symptoms.

	Day 8	3 months
Ex vivo cytokines		
TNFα	β = −0.04, *P* = 0.11	β = −0.01, *P* = 0.60
IL-1β	β = −0.05, *P* = 0.06	β = −0.02, *P* = 0.37
IL-6	β = −0.01, *P* = 0.42	β = −0.01, *P* = 0.50
IL-8	β = −0.01, *P* = 0.66	β = 0.03, *P* = 0.13
IL-12	β = 0.02, *P* = 0.90	β = −0.28, *P* = 0.21
IL-10	β = 0.01, *P* = 0.95	β = 0.18, *P* = 0.21
IP-10	β = −0.07, *P* = 0.05	β = −0.04, *P* = 0.28
Plasma cytokines		
IL-6	β = 0.24, *P* = 0.14	β = 0.42, *P* = 0.03 β = 0.36, *P* = 0.05^a^
TNFα	β = −1.04, *P* = 0.30	β = 1.30, *P* = 0.28
IL-1ra	β = 0.03, *P* = 0.47	β = 0.04, *P* = 0.32
sIL-6R	β = −0.01, *P* = 0.54	β = 0.02, *P* = 0.10

^a^Model adjusted for the use of antidepressive medication.

### Gene expression profiling

After exclusion of samples with poor quality RNA, RNA-sequencing data were available for 145 and 136 patients who underwent assessment of depressive symptoms on Day 8 and at 3 months after stroke, respectively.

When the *P*-value threshold was below 0.05, the gene expression of some TLR4-mediated cytokines and chemokines differed between groups. For example, there was differential mRNA expression of C-X-C Motif Chemokine Ligand 3 (CXCL3) between groups on Day 8, and of CXCL3, CCL2, IL-10, IL-1ra, and IL-1β at 3 months. However, after correction for multiple testing, gene expression levels of cytokines and chemokines did not differ between groups (Supplementary Tables 2 and 3).

## Discussion

In our study, LPS-induced mRNA and protein expression of cytokines did not differ between patients with and without greater depressive symptoms after stroke.

Few studies have examined the association between TLR4-mediated cytokine synthesis and depressive disorders, and these studies yielded conflicting results. In one study, several individual LPS-stimulated inflammatory markers including IL-8, IL-18, TNFβ, monocyte chemoattractant protein-1, and macrophage inflammatory protein-1β were associated with an increased odds ratio for current depressive disorder^45^. However, after adjusting for health and lifestyle factors, only IL-8 remained significantly associated with depressive disorder. In older persons, a higher production of IL-1β preceded a greater increase of depressive symptoms, whereas IL-1ra preceded a smaller increase of depressive symptoms^46^. Spijker et al. did not find any difference in the gene expression of LPS-stimulated cytokines between MDD patients and controls^47^.

Our results suggest that altered cytokine synthesis is not a predisposing factor for greater symptoms of PSD. Ex vivo released IL-1β and IP-10 predicted the severity of depressive symptoms assessed on Day 8. These associations were of borderline statistical significance. Our findings indicate that some LPS-induced cytokines may exert a weak effect on the severity of early depressive symptoms. These results need conformation in a larger population.

Our negative results do not exclude the possibility that impaired cytokine synthesis might occur not before but during the course of PSD as a consequence of mental disturbances. Furthermore, in contrast to blood-driven cytokines, TLR4-dependent cytokines that are synthesized by glia locally in the brain could contribute to the pathophysiology of PSD. Moreover, TLR4-independent mechanisms of cytokine synthesis by blood cells may be involved in the development of PSD symptoms.

In our study, the levels of measured plasma cytokines did not differ between groups, although a trend towards elevated IL-6 level was observed in patients with 3-month depressive symptoms. Moreover, circulating IL-6 predicted the severity of depressive symptoms assessed at 3 months after stroke.

Increased plasma levels of TNFα, IL-6, and IL-1ra have been found in patients with MDD^3–5,48^, but there have been conflicting results regarding circulating TNFα and IL-6 in PSD^18–21,23,24^. These studies have differed in methodology including diagnostic tools for depressive symptoms, inclusion of patients with prestroke depressive symptoms or taking antidepressive medication, the time of blood sampling, and the time between blood collection and assessment of depressive symptoms. Thus, direct comparison of their results is impossible.

Our results showing that circulating IL-6 might be associated with depressive symptoms at 3 months after stroke are in line with the results of meta-analyses showing that IL-6 is the most consistently elevated cytokine in the blood of patients with MDD^3,4^. Preclinical studies have demonstrated that peripheral IL-6 plays a role in the development of depression-like behavior^49^. Rodents exposed to different stressors display elevated circulating levels of IL-6 following the onset of depression-like behavior^50^. Moreover, increased IL-6 in the blood predisposes mice to developing depression-like symptoms after exposure to stress^51^. IL-6 knockout mice exhibit resistance to stress-induced depressive-like behavior^52^.

This study had several limitations. First, patient selection bias occurred, as so some patients with severe stroke or left-hemisphere lesions were excluded from the study. In these patients, depressive symptoms were untestable due to death, severe aphasia, consciousness disturbances (coma, stupor), or severe cognitive impairment. As a result, our cohort included mostly patients with milder stroke. Second, blood samples were only taken at a single time point. In contrast to ex vivo cytokine synthesis, which seems to be relatively stable during the first week after stroke, levels of circulating cytokines can change significantly^53^. Thus, the serial measurement of plasma cytokine would provide better insights into relationships between circulating inflammatory markers and PSD. Third, the diagnosis of greater depressive symptoms was based on the results of questionnaire instead of the psychiatric diagnosis. Levis et al. evaluated the diagnostic accuracy of PHQ-9 compared with major depression diagnosis from validated diagnostic interviews^54^. Meta-analysis performed by them has shown that the cutoff score of 10 or greater maximized combined sensitivity and specificity. Other studies have shown good reliability and validity of PHQ-9 in stroke patients^36,37^. In these studies, the cutoff score of 10 had sensitivity of 91–100% and sensitivity of 86–89% for the diagnosis of major depression. Fourth, infarct size was not measured in our study. Infarct volume is an important determiner of the post-stroke immune reaction^55^ and has been associated with post-stroke depression in some studies^56^. Fifth, prestroke cytokine levels are unknown in our study. Thus, prestroke rather than post-stroke IL-6 levels could be possibly related to depressive symptoms^57^. Finally, due to the limited number of patients, our exploratory study might be underpowered to demonstrate subtle associations between ex vivo synthesized cytokines and PSD.

To sum up, TLR4-dependent cytokine synthesis was not associated with greater symptoms of PSD in this study. Circulating IL-6 might be associated with depressive symptoms occurring at 3 months after stroke.

## Supplementary information

Supplementary Table 1

Supplementary Table 2

Supplementary Table 3
