# Supplementary material for: Toll-like receptor 4-mediated cytokine synthesis and post-stroke depressive symptoms
Source: Transl Psychiatry. 2021 Apr 26;11:246. doi: 10.1038/s41398-021-01359-x (PMC8076201; doi:10.1038/s41398-021-01359-x)
Supplement: Supplementary file 1 — Supplementary Table 1 [file 41398_2021_1359_MOESM1_ESM.docx]

Supplementary Table 1. Cytokines synthesized ex vivo and circulating cytokines in male and female patients.

A. Men.

|  | Day 8 | | | 3 months | | |
| --- | --- | --- | --- | --- | --- | --- |
|  | High score of depressive symptoms (N=20) | Low score of depressive symptoms (N=84) | P value | High score of depressive symptoms (N=26) | Low score of depressive symptoms (N=64) | P value |
| *Ex vivo* cytokines  (pg/mL) |  |  |  |  |  |  |
| TNFα | 2701  (2007 - 3513) | 2781  (2023 - 4106) | 0.60 | 2806  (2103 - 4028) | 2799  (2227 - 4254) | 0.61 |
| IL-1β | 1817  (1013 - 2039) | 1749  (1247 -2472) | 0.17 | 1761  (1095 - 2747) | 1898  (1208 - 2541) | 0.36 |
| IL-6 | 12356  (9264 - 19330) | 13631  (9682 - 17706) | 0.59 | 11396  (9127 - 18466) | 13994  (10615 - 18229) | 0.38 |
| IL-8 | 1403  (921 - 2255) | 1708  (1061 - 2446) | 0.45 | 2108  (1196 - 2918) | 1507  (939 - 2264) | 0.09 |
| IL-12 | 6.8 (1.1 – 13.7) | 4.6 (0.7 – 10.4) | 0.49 | 4.0 (2.1 – 8.2) | 6.4 (1.0 – 13.4) | 0.33 |
| IL-10 | 44.5 (34.6 – 56.6) | 53.8 (32.0 – 72.7) | 0.28 | 45.5 (32.8 – 82.5) | 47.5 (32.0 – 69.4) | 0.52 |
| IP-10 | 562 (278 - 834) | 528 (303 - 915) | 0.90 | 409 (208 - 763) | 543 (351 - 843) | 0.13 |
| Plasma cytokines  (pg/mL) |  |  |  |  |  |  |
| IL-6 | 5.8 (2.9 - 14.7) | 3.3 (1.8 – 6.7) | 0.06 | 4.9 (2.3 – 8.2) | 3.0 (1.5 – 6.6) | 0.07 |
| TNFα* | 0.7 (0.4 – 1.0) | 0.6 (0.4 – 1.0) | 0.99 | 0.6 (0.5 – 1.0) | 0.6 (0.3 – 1.3) | 0.68 |
| IL-1ra** | 587 (269 - 803) | 512 (367 - 731) | 0.65 | 472 (349 - 656) | 514 (338 - 703) | 0.84 |
| sIL-6R*** | 32400  (28200 - 41600) | 33000  (27400 - 37500) | 0.99 | 31750  (27000 - 39400) | 32000  (27200 - 35100) | 0.48 |

*samples available for 72 (12/60) patients and 65 (18/47) patients who were examined on Day 8 and 3 months after stroke, respectively

** samples available for 75 (14/61) patients and 83 (26/57) patients who were examined on Day 8 and 3 months after stroke, respectively

*** samples available for 82 (15/67) patients and 83 (26/57) patients who were examined on Day 8 and 3 months after stroke, respectively

B. Women.

|  | Day 8 | | | 3 months | | |
| --- | --- | --- | --- | --- | --- | --- |
|  | High score of depressive symptoms (N=7) | Low score of depressive symptoms (N=59) | P value | High score of depressive symptoms (N=11) | Low score of depressive symptoms (N=45) | P value |
| *Ex vivo* cytokines  (pg/mL) |  |  |  |  |  |  |
| TNFα | 1298  (1267 - 2757) | 2008  (1315 - 3199) | 0.49 | 1928  (1289- 2722) | 2279  (1555 - 3562) | 0.25 |
| IL-1β | 1252  (879 - 1533) | 1331  (998 - 1780) | 0.50 | 1331  (978 - 1536) | 1436  (899 - 2204) | 0.36 |
| IL-6 | 8000  (7338 - 11485) | 9205  (6701 - 13570) | 0.74 | 8001  (6363 - 13319) | 10319  (7753 - 13517) | 0.42 |
| IL-8 | 1339  (1084 - 1861) | 1607  (987 - 2653) | 0.66 | 1968  (781 - 2381) | 1528  (1028 - 2653) | 0.99 |
| IL-12 | 5.1 (0 – 7.3) | 3.4 (0 – 6.3) | 0.45 | 3.3 (2.4 – 6.1) | 4.2 (0.5 – 7.0) | 0.76 |
| IL-10 | 45.9 (36.3 – 83.6) | 44.9 (30.2 – 78.8) | 0.52 | 54.9 (36.3 – 78.1) | 44.9 (32.0 – 76.2) | 0.66 |
| IP-10 | 303 (176 - 635) | 330 (163 - 627) | 0.85 | 307 (125 - 627) | 356 (191 - 714) | 0.30 |
| Plasma cytokines  (pg/mL) |  |  |  |  |  |  |
| IL-6 | 2.0 (1.4 – 42.0) | 4.0 (2.3 – 14.6) | 0.63 | 4.2 (2.9 – 39.8) | 3.5 (2.2 – 7.3) | 0.29 |
| TNFα* | 0.6 (0.4 – 0.7) | 0.8 (0.4 – 1.2) | 0.50 | 0.8 (0.4 – 1.3) | 0.8 (0.4 – 1.0) | 0.50 |
| IL-1ra** | 386 (364 - 943) | 700 (356 - 970) | 0.96 | 763 (374 - 1330) | 785 (427 - 1080) | 0.81 |
| sIL-6R*** | 25900  (23000 - 28700) | 31650  (24200 - 38400) | 0.13 | 33600  (24200 - 40900) | 29900  (25400 - 38400) | 0.54 |

*samples available for 51 (5/46) patients and 41 (9/32) patients who were examined on Day 8 and 3 months after stroke, respectively

** samples available for 52 (5/47) patients and 51 (10/41) patients who were examined on Day 8 and 3 months after stroke, respectively

*** samples available for 65 (6/50) patients and 51 (10/41) patients who were examined on Day 8 and 3 months after stroke, respectively
